# ‘Why genes in pieces?’—revisited

**DOI:** 10.1093/nar/gkz284

**Published:** 2019-04-18

**Authors:** Ben Smithers, Matt Oates, Julian Gough

**Affiliations:** 1 Department of Computer Science, University of Bristol, Bristol BS8 1UB, UK; 2 MRC Laboratory of Molecular Biology, Cambridge CB2 0QH, UK

## Abstract

The alignment between the boundaries of protein domains and the boundaries of exons could provide evidence for the evolution of proteins via domain shuffling, but literature in the field has so far struggled to conclusively show this. Here, on larger data sets than previously possible, we do finally show that this phenomenon is indisputably found widely across the eukaryotic tree. In contrast, the alignment between exons and the boundaries of intrinsically disordered regions of proteins is not a general property of eukaryotes. Most interesting of all is the discovery that domain–exon alignment is much more common in recently evolved protein sequences than older ones.

## INTRODUCTION

In 1978, Walter Gilbert asked: ‘Why genes in pieces?’ (1). This question was posed shortly after the discovery of the intron–exon architecture of eukaryotic genes. It was hypothesized that exons should correspond to some unit of protein sequence, thus allowing rapid evolution of new proteins and new functions through the shuffling of exons (1,2). Early evidence of this were limited and contradictory, with example followed by counterexample. Indeed, even the same data were used to argue both for and against the idea (3–6). More recent large-scale studies have however found some support for the idea, by examining exon shuffling in the context of domains—which are units of proteins that can evolve, fold and function independently. Domains may typically be encoded by multiple exons, but the boundaries of exons have now been shown to align with the boundaries of domains more often than random (7). Additionally, a number of studies have analysed the phase of introns that flank domains, finding elevated levels of phase symmetry and in particular a strong increase in the use of phase 1-1 exons in metazoan genomes (8–10). Whilst it is not the case that all domains align with exon boundaries (symmetric or otherwise), which hampered the earliest attempts to determine such a correspondence, these more recent studies do show an overall trend. With the wealth of genome sequences now available, we establish this more universally and compare the phenomenon in newly evolved protein sequences with older protein sequences. In addition, we consider whether exon boundaries align with predicted regions of intrinsic disorder, which do not fold into a single, stable structure under natural conditions. Since alternatively spliced exons show enrichment for protein disorder (11–13), a correspondence between exon boundaries and regions of disorder may be expected.

## MATERIALS AND METHODS

### Data set

The loci of exons for all transcripts of 91 eukaryotic genomes were extracted from the Ensembl database (version 63 for genomes taken from the main Ensembl project; version 16 for those taken from Ensembl Fungi and Ensembl Plants; version 17 for those taken from Ensembl Metazoa and Ensembl Protists) (14). Genomes were selected on the basis of those available in both the SUPERFAMILY and D^2^P^2^ databases (15,16). The coordinates of each exon were mapped to protein sequence positions. Domain annotations were extracted directly from the SUPERFAMILY database. Disorder annotations were provided by D2P2 consensus disorder—a residue is considered disordered if at least 75% of the individual predictors within D^2^P^2^ predict it to be disordered. Finally, the data set was filtered to proteins that are encoded by at least two exons; three genomes (Saccharomyces cerevisiae, Leishmania major and Cyanidioschyzon merolae) were excluded from the analysis as they contained so few multi-exon transcripts.

### Aligning exon boundaries with domain and disorder boundaries

To determine if exon boundaries align with domain boundaries, a similar method to that used by Liu and Grigoriev was used (7). A window of residues was defined around the start and end of each domain assignment, to include one residue either side of the start and end of the domain. Thus for each domain, a total of six residues are considered to correspond to the domain boundary. For each protein sequence, we then counted the total number of internal exon boundaries (i.e. excluding the start of the first exon and the end of the last) that fall within any domain boundary window. For each genome, this was summed across all protein sequences that contained at least one domain, giving the observed number of exon boundaries aligning with domains. The number of exon boundaries expected to align is determined assuming they are distributed randomly throughout the protein sequence by multiplying the proportion of a protein’s residues found within any domain boundary window by the number of exon boundaries. This procedure was repeated similarly for the boundaries of predicted disordered regions.

### Comparing domain–exon alignment in old and new proteins

Using SUPERFAMILY’s ancestral reconstructions of domain content, novel domain architectures that are most likely to have been created at each genome’s node in the species tree were identified (17). Proteins with such novel architectures were considered ‘new’, all other proteins in each genome formed the set of ‘old’ proteins. Four genomes were excluded from this analysis as they contained very few proteins with novel domain architectures (Felis catus, Schizosaccharomyces pombe, Otolemur garnettii, Ictidomys tridecemlineatus).

### Statistical tests

**Figure 1.**
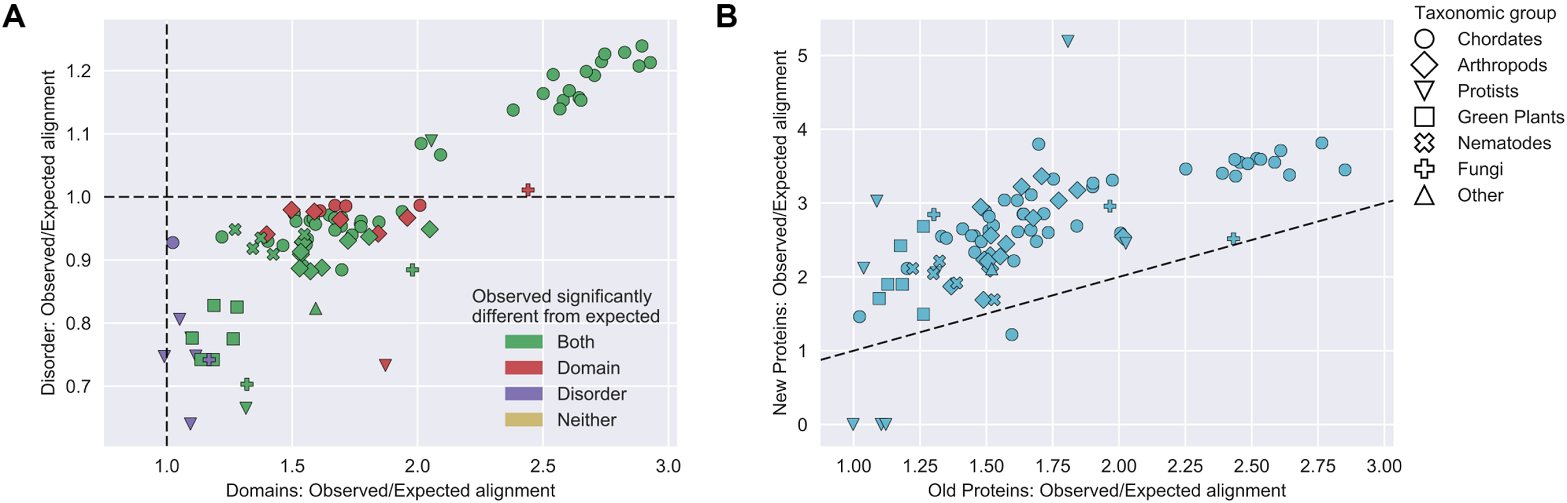
Ratios of observed to expected numbers of exon boundaries aligning to boundaries of domain and disorder assignments in 88 eukaryotic genomes. The shape of each point shows the taxonomic group. Within the legend, groups are ordered by the number of genomes they contain. (**A**) Observed/expected ratios for domain assignments on the *x*-axis; for disorder assignments on the *y*-axis. Dotted lines highlight where observed = expected. Colours indicate whether the difference in observed and expected numbers is significant (*P* < 0.01). (**B**) Observed/expected ratios for domain assignments calculated on proteins with novel domain architectures (*y*-axis) and all other proteins (*x*-axis). Dotted line corresponds to *y* = *x*, where the ratio is the same in new and old proteins.

To test the significance of the difference in observed and expected numbers of exon boundaries aligning to domain boundaries (and similarly for disordered regions), a chi-square test was used in line with previous analyses (7). To determine the significance of the difference in domain–exon alignment in new and old proteins, a bootstrap test was applied. We randomly partitioned each genome’s protein sequences into two sets (of the same size as the new and old protein sets defined above) 50 000 times. For each trial, we counted the number of genomes having a larger observed/expected ratio of domain–exon alignment in the smaller set (i.e. would be placed above the line in Figure 1B). The proportion of trials where this is true for at least as many genomes as in the new and old sets of proteins gives the significance of domain–exon alignment being greater in newly evolved proteins.

## RESULTS

For 88 eukaryote genomes, we counted the number of exon boundaries that, when mapped to protein sequence positions, are within one residue of the start or end of a SUPERFAMILY structural domain assignment or a D^2^P^2^ disorder assignment. Expected frequencies were calculated from the null hypothesis of exon boundaries being randomly distributed within a protein sequence.

### Exons can align with domain boundaries

Domain–exon alignment occurs more than expected in 87 of 88 genomes in our study. Figure 1A shows all but one of the points (each representing a genome) to the right of 1.0 on the *x*-axis. The *x*-axis is the ratio between the observed and expected number of exon boundaries that align to domain assignments. In genomes where the ratio is statistically significant for that genome alone, points are shown in red or green. The genomes with the greatest domain–exon alignment are all chordates, though as this is a large grouping there is large range, with exons aligning to domains between 1.5*x* and 3*x* as often as is expected by chance in most of these genomes. In addition, a significant correspondence between exon boundaries and domain boundaries is observed throughout the plants, nematodes and arthropods in this study, as well as some fungi and protists. Those genomes that do not display a significant alignment between domains and exons typically have comparatively few multi-exon transcripts. Taken together, it is clear that the alignment between exon boundaries and domain boundaries is a general property observed in eukaryotic genomes.

### Exons don’t align with disorder boundaries

For protein disorder, a different picture emerges when considering the distribution of genomes over the *y*-axis, which shows the alignment of exons to regions of predicted disorder. Though the boundaries of disordered regions do align more than expected in certain genomes (above the 1.0 line), they align less than expected in many others (below the 1.0 line). Domain and disorder boundary ratios are not independent, as seen by the points clustering on a diagonal line (Pearson *R* = 0.89; *P* < 1.2E-30). This is not surprising as disordered regions can share a boundary with a structural domain. Importantly, the regions of predicted disorder do not necessarily reflect conserved protein sequence. It may be that such conserved disorder—sometimes termed disordered domains (18,19)—has a similar relationship with exon boundaries as globular domains, but we have not tested that here. There do not appear to be any obvious taxonomic differences in disorder-exon alignment, beyond that due to the correlation with domain–exon alignment. D^2^P^2^ provides a consensus of many disorder predictors, but we find similar results are obtained when considering the individual predictors, thus the results are not an artefact of the consensus predictor or the peculiarities of any individual predictor.

### Exons align with boundaries more often in recently evolved proteins

Returning to exons in structured domains and considering their evolution, we found that boundaries correlate more often in recently evolved proteins than in older proteins. For each genome, we examined those proteins that have undergone a domain re-arrangement since the last ancestor common to another sequenced genome (using SUPERFAMILY ancestral reconstruction (17)). These proteins have a unique domain architecture that is not seen in other evolutionarily related genomes, and we call these ‘new’ as opposed to proteins whose architecture is shared with other genomes in the evolutionary clade, which we call ‘old’. Figure 1B shows the ratio between observed and expected domain–exon alignment for older proteins on the *x*-axis and new proteins on the *y*-axis. Since most genomes are above the dotted line of *y* = *x*, we can see that domain–exon alignment occurs more frequently for these new proteins than in all other proteins (*P* < 0.0001). For example, in the case of the human genome, there are 304 proteins identified as containing novel domain architectures. Exons in these proteins align to the boundaries of domains more than 3.2*x* as often as expected by chance, compared to 1.9*x* as often in all other proteins in the human genome. The relative difference between new and old proteins appears fairly consistent in the different taxonomic groups.

## DISCUSSION

In summary, we have shown that the boundaries of exons align with the boundaries of domains more than expected by chance and that this effect is stronger in recently evolved proteins. This is found to be a consistent property of eukaryotic genomes and provides strong evidence that exon shuffling has played some role in the evolution of novel domain architectures throughout eukarya. The alignment of exon boundaries with boundaries of disordered regions is, in contrast, variable and inconsistent. It remains to be seen whether conserved regions of intrinsic disorder display a similar relationship with exon boundaries as globular domains.

It is now clear, although previously suspected, that genes-in-pieces facilitates the modular evolution of the proteome, as well as affording the diversity and complexity obtained through alternative splicing. Nonetheless, we wish to highlight a more specific question: why domains in pieces? Many domains are encoded over multiple exons, yet the reuse of parts of a domain through shuffling and splicing ought not to be evolutionarily beneficial if domains act as units of protein sequence, structure or evolution. As a starting point for exploring this deeper question, we provide an interactive website for visualizing the locations of splice junctions on structured domains. This can be found at http://supfam.mrc-lmb.cam.ac.uk/exons

## DATA AVAILABILITY

The interactive website for visualizing the locations of splice junctions on structured domains is available at http://supfam.mrc-lmb.cam.ac.uk/exons.
